# Double Trouble: COVID-19 Infection Exacerbates Sickle Cell Crisis Outcomes in Hospitalized Patients—Insights from National Inpatient Sample 2020

**DOI:** 10.3390/hematolrep16030041

**Published:** 2024-06-29

**Authors:** Zubair Hassan Bodla, Mariam Hashmi, Fatima Niaz, Austin B. Auyeung, Anuoluwa Oyetoran, Muhammad Jahanzeb Khalil, Muhammad Salman Faisal, Farhan Khalid, Abdel-Rahman Zakieh, Yvette Bazikian, Christopher L. Bray

**Affiliations:** 1 Department of Internal medicine, Graduate Medical Education, College of Medicine, University of Central Florida (UCF), Orlando, FL 32827, USA; 2 Internal Medicine Residency Program, HCA Florida North Florida Hospital, Gainesville, FL 33328, USA; 3 Department of Internal Medicine, King Edward Medical University, Lahore 54000, Punjab, Pakistan; 4 Department of Internal Medicine, University of Alabama, Montgomery, AL 36116, USA; 5 Roswell Park Comprehensive Cancer Center, Buffalo, NY 14263, USA; 6 Department of Internal Medicine, Monmouth Medical Center, Long Branch, NJ 07740, USA

**Keywords:** sickle cell crisis, COVID-19 complications, National Inpatient Sample (NIS), healthcare resource utilization, inpatient mortality

## Abstract

**Background:** This study investigated the impact of COVID-19 on patients with sickle cell crisis (SCC) using National Inpatient Sample (NIS) data for the year 2020. **Methods:** A retrospective cohort analysis was conducted utilizing International Classification of Diseases (ICD-10) codes to identify adults who were admitted with a principal diagnosis of sickle cell crisis. The primary outcomes examined were inpatient mortality, while the secondary outcomes assessed included morbidity, hospital length of stay, and resource utilization. Analyses were conducted with STATA. Multivariate logistic and linear regression analyses were used to adjust for confounding variables. **Results:** Of 66,415 adult patients with a primary SCC diagnosis, 875 were identified with a secondary diagnosis of COVID-19 infection. Unadjusted mortality rate was higher for SCC patients with COVID-19 (2.28%) compared to those without (0.33%), with an adjusted odds ratio (aOR) of 8.49 (*p* = 0.001). They also showed increased odds of developing acute respiratory failure (aOR = 2.37, *p* = 0.003) and acute kidney injury requiring dialysis (aOR = 8.66, *p* = 0.034). Additionally, these patients had longer hospital stays by an adjusted mean of 3.30 days (*p* < 0.001) and incurred higher hospitalization charges by an adjusted mean of USD 35,578 (*p* = 0.005). **Conclusions:** The SCC patients with COVID-19 presented higher mortality rates, increased morbidity indicators, longer hospital stays, and substantial economic burdens.

## 1. Background

Sickle cell disease (SCD) is a hereditary hematologic disorder characterized by the presence of abnormal hemoglobin caused by a missense mutation in the beta-globin gene, leading to the production of rigid, sickle-shaped red blood cells [1,2]. This structural abnormality renders the red blood cells prone to deformation in low-oxygen environments, causing vaso-occlusion in small blood vessels and resulting in tissue ischemia and subsequent organ damage [3]. The clinical spectrum of SCD encompasses a wide range of acute complications due to vaso-occlusive crises, such as sickle cell crisis (SCC), which collectively contribute to the substantial morbidity and early mortality experienced by affected individuals [4,5].

One of the defining features of SCD is a state of chronic inflammation and oxidative stress due to recurrent hemolysis and endothelial dysfunction, further exacerbating the susceptibility to infections [6]. Furthermore, a primary distinguishing characteristic of SCD is functional asplenia, which confers upon those affected with a lifelong vulnerability to infections, especially those caused by encapsulated bacteria like Streptococcus pneumoniae and Haemophilus influenzae [4]. This dual burden of chronic inflammation and functional asplenia underscores the compromised immune state of SCD, heightening their risk of infections. Research studies have emphasized the significant role of infections in SCD-related mortality, with estimates suggesting that infections contribute to approximately 22% of SCD-related deaths in the United States [4,7].

Against this backdrop, the emergence of the global COVID-19 pandemic caused by the novel coronavirus SARS-CoV-2 in late 2019 has raised critical concerns for individuals with SCD [8]. COVID-19, characterized by a range of clinical manifestations, including mild respiratory symptoms to severe respiratory distress, multi-organ failure, and death, has presented a substantial threat to public health [9].

In the context of COVID-19, concerns have risen regarding the susceptibility of SSD patients to this novel virus. Viral infections, especially respiratory ones, are known precipitants of SCC [10], and they can further exacerbate the severity of SCC’s presentation. The interplay between SCC and COVID-19 is crucial to understand, given the associated heightened risk. The compromised immune systems, chronic inflammatory state, and damaged organs among the patients with sickle cell disease and vaso-occlusive pain crisis put them at an increased risk of severe COVID-19 disease. 

Utilizing data from the 2020 National Inpatient Sample (NIS), this study aims to explore the impact of COVID-19 on patients hospitalized primarily due to an episode of SCC. The insights drawn from this investigation could have far-reaching implications for patient care, providing directions for clinicians handling this delicate intersection of SCC and COVID-19. Such insights are essential for guiding clinical management, improving patient care, and informing public health strategies for this high-risk population.

## 2. Objective

The main objective of our study was to examine how COVID-19 impacts the clinical outcomes of patients who were admitted to hospital with a primary diagnosis of sickle cell crisis.

## 3. Materials and Methods

### 3.1. Study Design and Database Description

Utilizing the 2020 National Inpatient Sample (NIS) dataset, we conducted a retrospective cohort analysis of adult patients admitted with a primary diagnosis of sickle cell crisis in the United States. The NIS 2020 dataset specifically covers hospitalizations within the period from 1 January 2020 to 31 December 2020. It derives from the State Inpatient Databases (SIDs) that cover an estimated 98% of the U.S. population. As part of the Healthcare Cost and Utilization Project sponsored by Agency for Healthcare Research and Quality (AHRQ), the NIS is the most extensive, de-identified, publicly available inpatient database in the U.S. This database employs a 20% stratified discharge sample, which is then weighted relative to the total discharges to ensure national representativeness.

### 3.2. Study Patients

Patients hospitalized with a primary diagnosis of sickle cell crisis were identified using the International Classification of Diseases, Tenth Revision, with Clinical Modification (ICD-10-CM) codes. These codes have been implemented in the United States since October 2015. To ensure consistency and enable a more precise assessment of the effect of COVID-19, only those who were hospitalized with a principal diagnosis of sickle cell crisis were included in the analysis. The participants were divided into two groups based on whether they had a secondary diagnosis of COVID-19. The study excluded individuals who were under the age of 18. The ICD-10-CM and ICD-10-PCS codes are mentioned in the Supplementary Materials Table S1 and S2 respectively.

### 3.3. Study Variables and Outcomes

The primary outcome was all-cause inpatient mortality, while the secondary outcomes included the length of stay (LOS) in the hospital, resource utilization (such as the mean hospital charge and cost), and morbidity indicators, including acute respiratory failure, use of invasive and non-invasive ventilation, and acute kidney injury (AKI) that required dialysis. The study considered potential confounding variables that were collected and adjusted for, including characteristics of both the hospitals and the patients, including various clinically relevant comorbidities.

### 3.4. Statistical Analyses

Analyses were performed using STATA, version MP 14.2 (StataCorp, College Station, TX, USA). The study used univariate and multivariate regression analyses to compute unadjusted and adjusted outcomes, respectively. The univariate analysis was conducted to identify potential confounding factors. All confounders that displayed a significant relationship with the outcome, using a *p*-value threshold of 0.2 [11], were integrated into the multivariate model. Furthermore, variables deemed clinically relevant to the outcome from previous studies were included in the model, irrespective of their significance in the univariate analysis. Multivariate logistic regression analysis was carried out to determine the adjusted odds ratio, while multivariate linear regression analysis was used to find the adjusted mean values. Fisher’s exact test for proportions and Student’s *t*-test for continuous variables were utilized for other calculations. All *p*-values were two-sided, with a threshold of 0.05 being used for determining statistical significance. 

## 4. Results

### Patient Characteristics

Out of 32,355,827 weighted discharges in the 2020 NIS, only 76,890 (0.24%) patients had a primary diagnosis of sickle cell crisis, and of these patients, 66,415 were aged 18 or older, in line with our study’s inclusion criteria. Out of the patients diagnosed with sickle cell crisis (SCC), 65,540 were without a concurrent COVID-19 diagnosis, while 875 had both SCC and COVID-19. The average age for the SCC without COVID-19 group was 32.50 years, marginally higher than the 32.37 years observed for the SCC with COVID-19 group. Females represented 54.01% of the SCC without COVID-19 cohort and a slightly higher proportion, 58.86%, in the SCC with COVID-19 group (*p* = 0.219). The racial distributions between the groups were similar, with the Black population being the predominant group in both: 94.11% in the SCC without COVID-19 group and 93.14% in the SCC with COVID-19 group. However, these racial differences were not statistically significant (*p* = 0.709). The majority of the patients in both groups have a Charlson Comorbidity Index of 1, without any statistically significant difference between them (*p* = 0.775).

In terms of median household income, distributions across quartiles between the two groups were closely aligned. The largest distinction was found in hospital region distribution, with a significantly higher percentage of SCC with COVID-19 patients being from the northeast (28.0%) compared to the SCC without COVID-19 group (19.1%) (*p* = 0.029). A noticeable difference was also evident in hospital bed size, with a larger proportion of the SCC with COVID-19 patients (64.57%) being treated in large-bed hospitals compared to the SCC without COVID-19 group (55.07%) (*p*-value of 0.0105). The insurance, hospital location, and admission day categories showed no statistically significant differences between the groups. For instance, 72.01% of the SCC without COVID-19 patients were admitted on weekdays, compared to 76.57% of the SCC with COVID-19 patients (*p* = 0.146). 

The analysis of comorbidities in sickle cell crisis (SCC) patients with and without COVID-19 infection revealed varied prevalence rates, none of which achieved statistical significance. For example, COPD was observed in 2.46% of patients without COVID-19 compared to 2.29% of patients with the virus. Similarly, chronic kidney disease (CKD) affected 7.38% of non-infected patients and 5.71% of those infected. The smoking and Coronary Artery Disease (CAD) percentages showed a near-significant change, with smoking decreasing from 27.69% in non-infected patients to 21.14% in infected patients and CAD increasing from 1.58% to 3.43% as presented in Table 1.

## 5. Primary Outcome

### Mortality

The unadjusted inpatient mortality was notably different between the two cohorts studied. For SCC patients without COVID-19, mortality was observed in 220 patients, which translated into an unadjusted rate of 0.33%. In contrast, for SCC patients with COVID-19, mortality was documented in 20 patients, corresponding to an unadjusted rate of 2.28%. After adjusting for potential confounders using multivariate logistic regression analysis, SCC patients with COVID-19 exhibited a significantly higher adjusted mortality rate, with an adjusted odds ratio of 8.49 (95% CI: 2.27–31.68) and a *p*-value of 0.001 for r SCC patients with COVID-19 relative to those without it (as shown in Table 2).

## 6. Secondary Outcomes

### 6.1. Morbidity

Patients diagnosed with sickle cell crisis (SCC) and concomitant COVID-19 showed increased odds of developing acute respiratory failure and acute kidney injury that necessitated dialysis. Additionally, these patients had a heightened probability of needing non-invasive positive pressure ventilation. The overall incidence of acute respiratory failure was 3.70% for SCC patients without COVID-19 and 8.00% for those with the viral infection. Following our multivariate logistic regression analysis that accounted for various hospital and patient-related factors, the odds of experiencing acute respiratory failure were found to be notably greater in the COVID-19 group, with an adjusted odds ratio of 2.37 (*p*-value 0.003).

The requirement for non-invasive positive pressure ventilation exhibited a similar trend, standing at 0.67% for patients with SCC but without the viral infection and 2.28% for those with it. The adjusted analysis showed significantly increased odds in the latter group (aOR = 2.92; *p*-value 0.030). However, in terms of invasive ventilation, even though there was an observed rate of 0.62% in the non-COVID-19 group and 2.28% in the COVID-19 group, this difference did not reach statistical significance (*p*-value 0.290). Regarding acute kidney injury requiring dialysis, the observed rate was 0.18% for SCC patients without COVID-19 and 1.14% for those with it. Post adjustment, the COVID-19 cohort had significantly greater odds for this outcome (aOR = 8.66; *p*-value 0.034), as presented in Table 3.

### 6.2. Hospital Length of Stay 

Regarding hospital length of stay, SCC patients without COVID-19 had an average hospital stay of 5.19 days, while SCC patients diagnosed with COVID-19 had a notably longer average stay of 8.51 days. The adjusted mean difference in the length of stay between the two groups was 3.30 days, with a 95% confidence interval ranging from 1.65 to 4.96 days. This difference was statistically significant, as indicated by a *p*-value of less than 0.001 (Table 4). 

### 6.3. Resource Utilization

Hospitalization charges and costs were employed as indicators of resource utilization. For patients with sickle cell crisis without COVID-19, the total mean hospitalization charge was USD 41,694, whereas those with SCC with COVID-19 incurred a substantially higher total mean hospitalization charge of USD 77,344. After adjusting for potential confounding factors through a multivariate liner regression analysis, patients with sickle cell crisis and COVID-19 had a higher mean adjusted charge of USD 35,578 (95% CI; 10,923–60,234 USD; and *p*-value = 0.005). Similarly, the adjusted mean differential in costs between these two groups was USD 9605, captured within a 95% CI (USD 3268 to USD 15,942), with a *p*-value of 0.003 (Table 4). 

## 7. Discussion

Our study showed a significant increase in inpatient mortality, morbidity, and resource utilization among patients with sickle cell crisis (SCC) and secondary COVID-19 infection. Previous studies have primarily focused on comparing COVID-19 patients with and without sickle cell disease (SCD). A meta-analysis of 71 studies showed a higher mortality rate among those with SCD and COVID-19 [8], while a French study found no significant effect [12]. However, our study is distinct in that it is the first to assess the outcomes of an acute episode of sickle cell crisis complicated by COVID-19 using nationwide data.

The primary outcome measure, odds ratio of patient mortality, was markedly increased by 8.5-fold in SCC patients who also had COVID-19. This could be the result of compounded adverse effects, common in both conditions, leading to multi-organ failure. Coagulability has been found to be increased in both SCD and COVID-19 [13,14]. The degree of coagulability in patients with both conditions versus those with SCC or COVID-19 alone is not known. However, it is plausible that the likelihood of adverse events relating to hypercoagulability may be enhanced, resulting in increased cardiovascular events, stroke, and other ischemic complications, leading to significantly higher mortality. Moreover, the immune response to COVID-19 is known to be vigorous and can result in a “cytokine storm”, leading to extensive inflammation and tissue injury [15]. Given that the immune profile in SCD patients is already somewhat altered [16], COVID-19 infection may potentiate an even more severe and damaging immune reaction in this population.

Our study found that there were increased odds of acute respiratory failure in the SCC and COVID-19 patients compared to the SCC only patients. Patients with SCC and COVID-19 are at an elevated risk of acute respiratory failure due to the combined respiratory effects of both conditions. SCC can lead to acute chest syndrome (ACS), marked by chest pain, fever, and lung issues often resulting from infections or vaso-occlusion [17]. COVID-19 predominantly affects the respiratory system, causing conditions ranging from mild pneumonia to severe ARDS [15]. The compromised oxygen exchange from sickled red blood cells, coupled with COVID-19’s impacts on the lungs, intensifies respiratory issues [18]. Combined lung injuries can overwhelm the lungs’ healing capacities, escalating the risk of respiratory failure [19]. The patients with sickle cell crisis concurrently diagnosed with COVID-19 exhibited notably poorer clinical outcomes, particularly a heightened use of non-mechanical positive pressure ventilation. Yet, despite observing a higher adjusted odds ratio for mechanical ventilation in the COVID-19 cohort, the discrepancy did not reach statistical significance. Several potential reasons could account for this observation, including the rarity of the event, clinical decision-making factors, overlap with other respiratory interventions, potential unadjusted confounders, and inherent data limitations. Further research is essential to elucidate this difference more comprehensively.

Our analysis shows that the odds of acute kidney injury requiring dialysis were significantly increased by >8.5-fold in those with SCC and COVID-19 versus the SCC only patients. Patients with SCC have a 15% reversible reduction in creatinine clearance compared to baseline and may also present with acute subclinical tubular injury despite normal creatinine levels [20]. Over one-quarter of hospitalized COVID-19 patients develop acute kidney injury [21]. Regional inflammation, endothelial injury, viral invasion, and microthrombi have been proposed as possible mechanisms of renal injury, the latter of which may be due to hypercoagulability (mentioned previously) [21]. 

Our study has some limitations. Because it was retrospective, we could not fully randomize exposure. However, by employing multivariate regression models that took into account various patient and hospital traits, as well as co-morbidities, we made an effort to reduce confounding. Still, even with these measures, there is a slight chance that residual confounding occurred. To identify patients with sickle cell crisis and COVID-19, we opted for ICD-10 codes rather than clinical measures. This might have resulted in diagnosis misclassification. Yet, the legitimacy of using ICD-10 codes for this purpose is backed by earlier studies that utilized HCUP data [22]. Furthermore, the lack of available laboratory values in the HCUP data meant we could not classify disease severity, pain levels, anemia, percentage of sickle hemoglobin (HbS), or the severity of COVID-19 infection. This limitation extends to the detailed examination of immune response mechanisms such as cytokine and chemokine profiles, which could offer valuable insights into the exacerbated outcomes observed in sickle cell crisis patients with COVID-19. However, we used the Charlson Comorbidity Index to account for the comorbidity burden. Our report covers the overall inpatient mortality of those diagnosed with sickle cell crisis with and without COVID-19 since the exact cause of death could not be calculated from the NIS database. 

Another limitation of our study is the inability to distinguish between asymptomatic COVID-19 cases due to the absence of specific ICD-10 codes and subjective data in the NIS, potentially leading to variability. Particularly at the pandemic’s onset, the low rate of testing among asymptomatic individuals likely resulted in an underrepresentation of COVID-19 cases, reflected in the presumed negatives. To improve the evidence and address this study’s constraints, more randomized control trials are necessary.

Our study also has several strengths. We utilized the NIS, which contains deidentified patient data from over 48 states. This makes our findings a true reflection of the patient population admitted to hospitals across the country, showcasing extensive external validity and generalizability. It also overcomes the limitations of single-center studies. Its national representation grants access to unique variables related to hospitals and patients, such as household earnings, hospital zones, and many other parameters not feasible for consideration in smaller studies.

## 8. Conclusions

Our study suggests that patients with sickle cell crisis and COVID-19 infection require heightened medical attention and prompt treatment to mitigate the risk of adverse outcomes. These results also highlight the importance of preventive measures to limit the spread of COVID-19 infection, particularly among high-risk populations, such as those with sickle cell disease.

## Figures and Tables

**Table 1:** Characteristics of patients.

Basic Characteristics	SCC without COVID (*n* = 65,540)	SCC with COVID (*n* = 875)	*p*-Value
Age (Mean)	32.50	32.37	
Female gender, (%)	54.01	58.86	0.219
Race, (%)			0.709
White	6.2	0.57	
Black	94.11	93.14	
Hispanic	3.57	3.43	
Others *	1.71	2.86	
Charlson Comorbidity Index (%)			0.775
1	64.4	66.67	
2	19.31	21.33	
3	8.62	6.67	
≥4	7.67	5.33	
Median Household income(quartile), (%)			0.699
1st (0–25th)	50.37	50.0	
2nd (25–50th)	23.26	20.35	
3rd (50–75th)	16.04	18.02	
4th (75–100th)	10.33	11.63	
Hospital region, (%)			0.029
Northeast	19.1	28.0	
Midwest	19.35	16.0	
South	53.2	50.86	
West	8.35	5.14	
Hospital bed size, (%)			0.011
Small	21.02	12.57	
Medium	23.91	22.86	
Large	55.07	64.57	
Teaching status, (%)			0.197
Non-Teaching	14.44	10.86	
Teaching	85.56	89.14	
Insurance category, (%)			0.524
Medicare	32.6	28.07	
Medicaid	47.39	48.54	
Private	16.18	18.13	
Uninsured	3.83	5.26	
Hospital Location, (%)			0.515
Rural	3.38	2.29	
Urban	96.62	97.71	
Admission Day, (%)			0.146
Weekday admission	72.01	76.57	
Weekend Admission	27.99	23.43	
Co-Morbidities, (%)			
COPD	2.46	2.29	0.883
CKD	7.38	5.71	0.399
CHF	5.84	7.43	0.363
H/O CVA with residual neurological deficits	2.03	0.57	0.174
Obesity	7.18	8.57	0.472
Anemia	2.46	1.71	0.523
Smoking	27.69	21.14	0.058
CAD	1.58	3.43	0.056
Hypertension	14.62	17.71	0.278
Hyperlipidemia	2.02	4.00	0.061
Anxiety	13.59	13.14	0.864
Depression	13.98	13.71	0.921

* Native Americans/Pacific Islanders/other races. Abbreviations: SCC, sickle cell crisis; COPD, chronic obstructive pulmonary disease; CKD, chronic kidney disease; CHF, congestive heart failure; H/O CVA, history of cerebrovascular accident; CAD; Coronary Artery Disease. (Further information about median household income, hospital region, and hospital bed size can be found in the Supplementary Materials Tables S3, S4 and S5 respectively).

**Table 2:** Primary outcome—mortality.

Outcomes	SCC Patients without COVID-19	SCC Patients with COVID-19	Adjusted Odds Ratio	*p* Value
Inpatient mortality (%)	220 0.33 (0.25–0.45)	20 2.28 (0.87–5.88)	8.49 (2.27–31.68)	0.001

Abbreviations: SCC, sickle cell crisis.

**Table 3:** Morbidity.

Outcomes	SCC Patients without COVID-19	SCC Patients with COVID-19	Adjusted Odds Ratio	*p* Value
Acute respiratory failure (%)	3.70 (3.35–4.08)	8.00 (4.78–13.09)	2.37 (1.33–4.21)	0.003
Non-invasive Positive pressure ventilation (%)	0.67 (0.52–0.86)	2.28 (0.87–5.87)	2.92 (1.11–7.69)	0.030
Invasive ventilation (%)	0.62 (0.50–0.77)	2.28 (0.87–5.87)	3.39 (0.76–15.14)	0.109
AKI requiring dialysis (%)	0.18 (0.12–0.27)	1.14 (0.29–4.36)	8.66 (1.18–63.44)	0.034

Abbreviations: SCC, sickle cell crisis; AKI, acute kidney injury.

**Table 4:** Length of stay and resource utilization.

Outcomes	SCC Patients without COVID-19	SCC Patients with COVID-19	Adjusted Mean Value	*p*-Value
Length of stay- (Days)	5.19 (5.05–5.32)	8.51 (6.85–10.17)	3.30 (1.65–4.96)	<0.001
Total mean hospitalization charge (USD)	41,694 (39592–43795)	77,344 (52333–102356)	35,578 (10923–60234)	0.005
Total mean hospitalization cost (USD)	10,077 (9611–10542)	19,697 (13295–26099)	9605 (3268–15942)	0.003

Abbreviations: SCC, sickle cell crisis; USD, United States Dollar.

## Data Availability

The data analyzed in the study are publicly available through the Healthcare Cost and Utilization Project (HCUP).

## Supplementary Materials

The following supporting information can be downloaded at: https://www.mdpi.com/article/10.3390/hematolrep16030041/s1, Table S1: Diseases and their corresponding ICD 10 CM codes; Table S2: Procedures and their ICD 10-PCS codes; Table S3: Median household income in USD; Table S4: Hospital Region. Table S5: Hospital bed size categories (In number of beds), by region.
