# Supplementary material for: Double Trouble: COVID-19 Infection Exacerbates Sickle Cell Crisis Outcomes in Hospitalized Patients—Insights from National Inpatient Sample 2020
Source: Hematol Rep. 2024 Jun 29;16(3):421–30. doi: 10.3390/hematolrep16030041 (PMC11270312; doi:10.3390/hematolrep16030041)
Supplement: Supplementary file 1 [file hematolrep-16-00041-s001.zip › hematolrep-2742786-supplementary.pdf]

Supplementary Materials

Table S1. Diseases and their corresponding ICD 10 CM codes.

Name of Disease	ICD 10 CM codes
Sickle cell crisis	D570, D5721, D5741, D5743, D5745, D5781
COVID-19	U071
Acute kidney injury	N17x
Acute respiratory failure	J690

Table S2. Procedures and their ICD 10-PCS codes.

Name of Procedure	ICD 10 procedure codes
Non-invasive positive pressure ventilation	5A09357, 5A09457, 5A09557
Mechanical ventilation	5A1935Z, 5A1945Z, 5A1955Z
Dialysis	5A1D70Z, 5A1D80Z, 5A1D90Z

Table S3. Median household income in USD.

Quartile	2020
**First Quartile**	1 - 49,999
**Second Quartile**	50,000 - 64,999
**Third Quartile**	65,000 - 85,999;
**Fourth Quartile**	86,000 +

Table S4. Hospital Region.

Hospital region	Name of US States
Northeast	Maine, New Hampshire, Vermont, Massachusetts, Rhode Island, Connecticut, New York, Pennsylvania, New Jersey,
Midwest	Wisconsin, Michigan, Illinois, Indiana, Ohio, Missouri, North Dakota, South Dakota, Nebraska, Kansas, Minnesota, Iowa
South	Delaware, Maryland, Virginia, West Virginia, North Carolina, South Carolina, Georgia, Florida, Kentucky, Tennessee, Mississippi, Oklahoma, Texas, Arkansas, Louisiana
West	Montana, Wyoming, Nevada, Utah, Colorado, Arizona, New Mexico, Alaska, Washington, Oregon, California, Hawaii

Table S5. Hospital bed size categories (In number of beds), by region.

Location and teaching status	Small	Medium	Large
NORTHEAST			
Rural	1-49	50-99	100+
Urban, Non- teaching	1-124	125-199	200+
Urban, teaching	1-249	250-424	425+
MIDWEST			
Rural	1-29	30-49	50+
Urban, Non- teaching	1-74	75-174	175+
Urban, teaching	1-249	250-374	375+
SOUTH			
Rural	1-39	40-74	75+
Urban, Non- teaching	1-99	100-199	200+
Urban, teaching	1-249	250-449	450+
WEST			
Rural	1-24	25-44	45+
Urban, Non- teaching	1-99	100-174	175+
Urban, teaching	1-199	200-324	325+
